# Baltimore School of Veterinary Science

**Published:** 1890-09

**Authors:** 


*Baltimore School of Veterinary Science.*—Dr. Reed was transferred from the chair of physiology and pathology in the medical school, having ceased to be a professor in the veterinary school. Dr. Emiul W. Gilan was elected in his place. Professor Ward has published a work on "La Grippe." Professor Ward has been appointed State Veterinarian *vice* Dr. Wray, who has gone to England for the Bureau of Animal Industry. Dr. P. B. Wilson is acting dean.

